# Potential Influence of *ADAM9* Genetic Variants and Expression Levels on the *EGFR* Mutation Status and Disease Progression in Patients with Lung Adenocarcinoma

**DOI:** 10.3390/ijms26104606

**Published:** 2025-05-11

**Authors:** Jer-Hwa Chang, Tsung-Ching Lai, Kuo-Hao Ho, Thomas Chang-Yao Tsao, Lun-Ching Chang, Shun-Fa Yang, Ming-Hsien Chien

**Affiliations:** 1 School of Respiratory Therapy, College of Medicine, Taipei Medical University, Taipei 11031, Taiwan; 2 Pulmonary Research Center, Wan Fang Hospital, Taipei Medical University, Taipei 11696, Taiwan; 3 Division of Pulmonary Medicine, Department of Internal Medicine, Wan Fang Hospital, Taipei Medical University, Taipei 11696, Taiwan; 4 Department of Biochemistry and Molecular Cell Biology, School of Medicine, College of Medicine, Taipei Medical University, Taipei 11031, Taiwan; 5 School of Medicine, Chung Shan Medical University, Taichung 40201, Taiwan; 6 Department of Internal Medicine, Chung Shan Medical University Hospital, Taichung 40201, Taiwan; 7 Department of Mathematical Sciences, Florida Atlantic University, Boca Raton, FL 33431, USA; 8 Institute of Medicine, Chung Shan Medical University, Taichung 40201, Taiwan; 9 Department of Medical Research, Chung Shan Medical University Hospital, Taichung 40201, Taiwan; 10 Graduate Institute of Clinical Medicine, College of Medicine, Taipei Medical University, Taipei 11031, Taiwan; 11 Traditional Herbal Medicine Research Center, Taipei Medical University Hospital, Taipei 110301, Taiwan; 12 TMU Research Center of Cancer Translational Medicine, Taipei Medical University, Taipei 11031, Taiwan

**Keywords:** single-nucleotide polymorphism, a disintegrin and metalloprotease 9, epidermal growth factor receptor, clinicopathologic progression, lung adenocarcinoma

## Abstract

Lung adenocarcinoma (LUAD) is driven by epidermal growth factor receptor (*EGFR*) mutations, making it a key therapeutic target. ADAM9, a member of the A disintegrin and metalloproteinase (ADAM) family, facilitates the release of growth factors and was implicated in activating the EGFR-mediated progression in several cancer types. In this study, we explored potential associations among *ADAM9* single-nucleotide polymorphisms (SNPs), the *EGFR* mutation status, and the clinicopathological progression of LUAD in a Taiwanese population. In total, 535 LUAD patients with various *EGFR* statuses were enrolled, and allelic distributions of *ADAM9* SNPs—located in promoter and intron regions, including rs78451751 (T/C), rs6474526 (T/G), rs7006414 (T/C), and rs10105311 (C/T)—were analyzed using a TaqMan allelic discrimination assay. We found that LUAD patients with at least one polymorphic G allele in *ADAM9* rs6474526 had a lower risk of developing *EGFR* mutations compared to those with the wild-type (WT) TT genotype. Furthermore, G-allele carriers (TG + GG) of rs6474526 were associated with an increased likelihood of developing larger tumors (T3 or T4), particularly among patients with mutant *EGFR*. Conversely, in patients with WT *EGFR*, carriers of the T allele in rs10105311 had a lower risk of progressing to advanced stages (stage III or IV). Among females or non-smokers, G-allele carriers of rs6474526 demonstrated a higher risk of advanced tumor stages and distant metastases. In clinical data from the Genotype-Tissue Expression (GTEx) database, individuals with the polymorphic T allele in rs6474526 showed reduced ADAM9 expression in lung and whole blood tissues. Screening the genotype of rs6474526 in a set of LUAD cell lines revealed that cells carrying at least one minor G allele exhibited higher ADAM9 levels compared to those with the TT genotype. Additionally, analyses using TCGA and CPTAC databases revealed elevated ADAM9 expression in LUAD specimens compared to normal tissues. Elevated protein levels were correlated with advanced T stages, pathological stages, and worse prognoses. In summary, our results suggest that *ADAM9* genetic variants of rs6474526 may affect ADAM9 expression and are associated with the *EGFR* mutation status. Both rs6474526 and rs10105311 were correlated with disease progression in LUAD patients. These variants could serve as potential biomarkers for predicting clinical outcomes.

## 1. Introduction

Lung cancer is the most common cancer worldwide and ranks among the top three leading causes of cancer-related mortality [1]. Non-small-cell lung cancer (NSCLC) accounts for about 85% of lung cancer cases, with lung adenocarcinoma (LUAD) being the most common subtype. Alterations in oncogenic driver genes are one of the important causes of NSCLC. The epidermal growth factor receptor (EGFR), a cell surface tyrosine kinase receptor, is commonly mutated in LUAD, especially in East Asians, females, and non-smokers [2,3,4]. The two most common somatic mutations in NSCLC, particularly in LUAD, are an in-frame deletion in exon 19 and the L858R point mutation in exon 21, which together account for about 85% of all *EGFR* mutations. These mutations enhance EGFR activity, driving tumor cell proliferation, angiogenesis, migration, and invasion, thereby accelerating tumor progression. Consequently, recent research indicated that LUAD patients with *EGFR* mutations are ideal candidates for treatment with EGFR tyrosine kinase inhibitors (TKIs) [4].

A genetic predisposition for lung cancer has recently garnered significant attention from thoracic oncologists and geneticists, driven by the increasing focus on lung cancer in non-smokers and the widespread adoption of somatic next-generation sequencing panels that encompass cancer susceptibility genes [5]. *EGFR* somatic mutations are present in 30–50% of East Asian NSCLC patients, compared to less than 20% in other ethnic groups. Notably, this high prevalence remains consistent among East Asians who migrate to other regions, suggesting that genetic factors, rather than geographic or environmental influences, play a key role in mutation development [6]. Single-nucleotide polymorphisms (SNPs), which are variations in the DNA sequence of a genome, can result in alterations in genes that impact the function of cellular proteins and enzymes [7,8]. Genome-wide association studies (GWASs) and fine-mapping analyses have identified numerous SNPs associated with LUAD susceptibility in East Asians, including rs137884934 in the *PIK3CB* gene, rs682888 in the *DTNB* gene, rs116863980 in the *PALM* gene, rs4268071 in the *GPR37* gene, and so on [9]. Furthermore, GWASs have pinpointed SNPs potentially linked to an increased risk of *EGFR* mutation-positive LUAD, such as rs2179920 in the *HLA-DPB1* gene and rs2495239 in the *FOXP4* gene [10].

A disintegrin and metalloproteinase 9 (ADAM9) is a type I transmembrane glycoprotein that is part of the ADAM family. Recent research highlighted its association with the progression of various cancers [11,12]. In LUAD, ADAM9 is notably overexpressed and was shown to promote tumor growth in xenograft models by regulating angiogenic factors such as vascular endothelial growth factor A, interleukin-8, and angiopoietin-2 [13,14]. Additionally, increased ADAM9 levels have been recognized as an independent prognostic marker for overall survival (OS) in stage I NSCLC patients who underwent standard lobectomy and mediastinal N2 lymph node dissection [15]. The metalloprotease domain of ADAM9 is capable of cleaving the precursor of heparin-binding EGF-like growth factor (HB-EGF), producing soluble HB-EGF [16]. This, in turn, activates EGFR signaling, which drives tumor growth and angiogenesis [17]. Notably, *ADAM9* knockdown was shown to reduce EGFR signaling and enhance sensitivity to EGFR inhibitors in LUAD cells [18]. Furthermore, ADAM9 was implicated in promoting LUAD cell metastasis by increasing tissue plasminogen activator activity and activating the EGFR, which stimulates the migration-promoting protein, CUB domain-containing protein 1 [18,19]. The functional roles and clinical significance of the ADAM9/EGFR axis in LUAD have been previously reported. Studies have shown a correlation between pneumonia and the development of EGFR mutations in lung cancer [20], while ADAM9 has been identified as a key player in lung acute or chronic inflammation [21]. However, the influence of *ADAM9* genetic variants on LUAD, regardless of *EGFR* mutation status, has yet to be explored. In this study, we investigated relationships between *ADAM9* SNPs and the *EGFR* mutation status, and their influence on the clinicopathological characteristics of LUAD in a Taiwanese population. Herein, four SNPs in the *ADAM9* gene were selected: two located in the intronic region (rs6474526 and rs78451751) and two in the promoter region (rs7006414 and rs10105311). These SNPs were chosen based on previous reports indicating their association with the risk, severity, or progression of cancers or Alzheimer’s disease, as well as their influence on *ADAM9* gene expression [22,23]. Our findings indicated that rs6474526 intronic SNPs are associated with susceptibility to *EGFR* mutations. Furthermore, both rs6474526 intronic SNPs and rs10105311 promoter SNPs show potential as predictive markers for disease progression in LUAD patients. Additionally, our results suggest that ADAM9-related pathways, such as EMT, may play a crucial role in driving LUAD progression.

## 2. Results

### 2.1. Demographic and Clinical Profiles of LUAD Patients with WT or Mutant EGFR

Table 1 presents comparisons of demographic and clinical profiles between LUAD patients with WT *EGFR* (219 patients) and those with mutant *EGFR* (316 patients). There was no significant difference in the age distribution between the two groups. However, compared to the WT *EGFR* group, the mutant *EGFR* group exhibited a higher proportion of females (66.8% vs. 38.8%, *p* < 0.001), a greater percentage of non-smokers (80.7% vs. 47.0%, *p* < 0.001), and a higher rate of well/moderately differentiated tumors (89.6% vs. 70.3%, *p* < 0.001). Tumor stage and TNM statuses did not significantly differ between the WT and mutant *EGFR* groups. Overall, the demographic and clinical profiles of LUAD patients with mutant *EGFR* in this study aligned with previously reported findings in Asian LUAD populations [24].

### 2.2. Distribution of ADAM9 Candidate SNPs (rs6474526, rs7006414, rs10105311, and rs78451751) Among LUAD Patients and Their Correlations with the EGFR Mutation Status

To investigate potential relationships between *ADAM9* SNPs and the risk of developing *EGFR* mutations, genotypes and allelic frequencies of four SNPs [rs6474526 (intron region, T>G), rs7006414 (promoter region, T>C), rs10105311 (promoter region, C>T), and rs78451751 (intron region, T>C)] were examined in LUAD patients with either WT or mutant *EGFR*. The most common genotypes for these SNPs in both the WT and mutant *EGFR* groups were TT for rs6474526, TT for rs7006414, CC for rs10105311, and TT for rs78451751. After adjusting for age, gender, and smoking status, the TG and TG + GG genotypes of *ADAM9* rs6474526 were significantly associated with a reduced likelihood of *EGFR* mutations (AOR: 0.477, 95% CI: 0.260–0.875, *p* = 0.017) (Table 2). In contrast, no significant associations were observed for rs7006414, rs10105311, or rs78451751 SNPs with the risk of *EGFR* mutations.

### 2.3. Associations Between ADAM9 Polymorphic Genotypes and Clinicopathological Features of LUAD Patients with WT or Mutant EGFR

Next, to examine the effects of *ADAM9* genetic polymorphisms on the clinicopathological features of LUAD, factors including primary tumor size, cancer stage, lymph node involvement, cell differentiation, and distant metastasis were assessed. Among the four *ADAM9* loci studied, LUAD patients who carried at least one minor allele (TG + GG) of rs6474526 had a significantly increased risk of being at an advanced T stage (T3 or T4) compared to those with the WT homozygote (TT) (OR = 2.046, 95% CI = 1.099–3.807; *p* = 0.022), as demonstrated in Table 3. However, no significant associations were observed with clinicopathological features examined for rs7006414, rs10105311, or rs78451751. Further subgroup analyses stratified LUAD patients based on the WT or mutant *EGFR* status to evaluate relationships between *ADAM9* SNPs and clinicopathological characteristics. Among patients with mutant *EGFR*, carriers of the *ADAM9* rs6474526 G allele (TG + GG) demonstrated a significantly increased risk of larger tumor sizes (OR = 2.939, 95% CI = 1.099–7.858; *p* = 0.025). In contrast, no significant associations between rs6474526 and clinicopathological statuses were found in patients with WT *EGFR* (Table 3). Additionally, the dominant model of *ADAM9* rs10105311 was significantly associated with a reduced risk of advanced clinical stages (OR = 0.485, 95% CI = 0.249–0.946; *p* = 0.032) in LUAD patients with WT *EGFR* (Table 4).

### 2.4. Associations Between ADAM9 Polymorphic Genotypes and Clinicopathological Characteristics in Female LUAD Patients and Non-Smoking LUAD Patients

Considering the link between rs6474526 and the occurrence of *EGFR* mutations in LUAD patients, along with the high prevalence of *EGFR* mutations in female never-smokers with LUAD in Asian populations [25], we further analyzed correlations between variant genotypes of *ADAM9* rs6474526 and clinical characteristics within female and non-smoking populations. Notably, rs6474526 variants (TG + GG) were significantly associated with larger tumor sizes and distant metastases in both the female and non-smoking groups (Table 5).

### 2.5. Potential Effects of ADAM9 Genetic Variants on ADAM9 Expression

**Figure 1:**
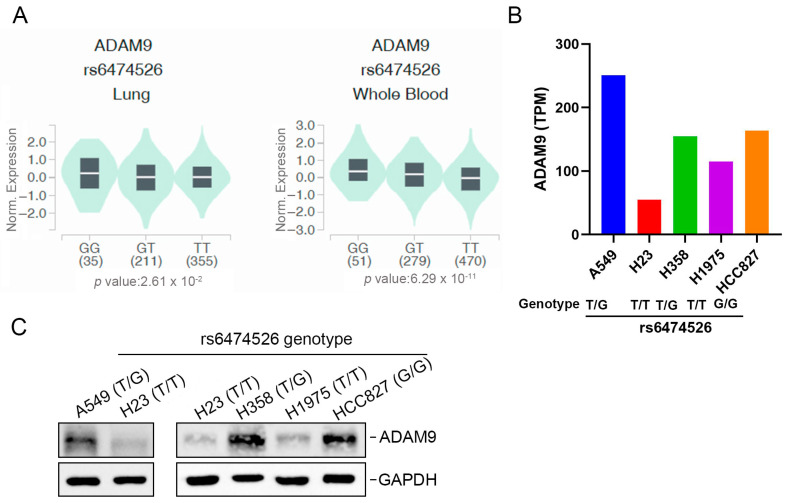
The effects of *ADAM9* rs6474526 polymorphisms on ADAM9 expression were evaluated using validated datasets from the Genotype-Tissue Expression (GTEx) portal (https://gtexportal.org/home/, accessed on 12 Dec 2024) and the DepMap portal (https://depmap.org/portal/, accessed on 16 Jan 2025). (**A**) Violin plots show that the G allele of rs6474526 was associated with higher *ADAM9* expression in lung tissues and whole blood. The Y-axis represents normalized *ADAM9* gene expression levels (Norm. Expression), which have been adjusted to minimize technical and biological variation, allowing accurate comparison across different genotypes. The *p*-value on the X-axis indicates the level of statistical significance for the association between the rs6474526 SNP and ADAM9 expression. (**B**) Relationships between *ADAM9* rs6474526 genotypes and ADAM9 expression levels were analyzed in five lung adenocarcinoma (LUAD) cell lines. The lower panel shows genotypes of *ADAM9* rs6474526 in LUAD cells (A549, H23, H358, H1975, and HCC827), determined using a TaqMan SNP Genotyping Assay, while the upper panel presents *ADAM9* mRNA expression levels obtained from the DepMap portal. (**C**) The ADAM9 protein expression levels were analyzed by the Western blot assay in LUAD cells carrying the wild-type or mutant genotype of ADAM9 rs6474526.

We further examined relationships between *ADAM9* genetic variants and *ADAM9* gene expression in healthy individuals’ whole blood and lung samples using data from the GTEx database. For rs6474526, individuals with the WT homozygous TT genotype displayed the lowest *ADAM9* gene expression levels in both lung tissues and whole blood compared to those carrying at least one minor allele (Figure 1A). We next investigated associations between rs6474526 genotypes and ADAM9 expression levels across various LUAD cell lines (A549, H358, H23, HCC827, and H1975). Our analysis revealed that A549, H358, and HCC827 cells carried the TG or GG mutant genotypes of rs6474526, whereas H23 and H1975 cells had the TT genotype (Figure 1B, lower panel). Using RNA expression data from the DepMap portal, we found that LUAD cell lines with at least one minor G allele (A549, H358, and HCC827) exhibited higher *ADAM9* gene expression levels compared to H23 and H1975 cells with the TT genotype (Figure 1B, upper panel). We further analyzed ADAM9 protein levels in these LUAD cell lines and observed higher ADAM9 expression in A549 cells compared to H23 cells (Figure 1C, left panel). Similarly, other LUAD cell lines (H358 and HCC827) carrying at least one minor G allele exhibited higher ADAM9 protein levels than LUAD cells (H23 and H1975) with the WT genotype (Figure 1C, right panel).

### 2.6. Associations Between ADAM9 Expression Levels and Clinicopathological Characteristics as Well as Prognoses of LUAD Patients

**Figure 2:**
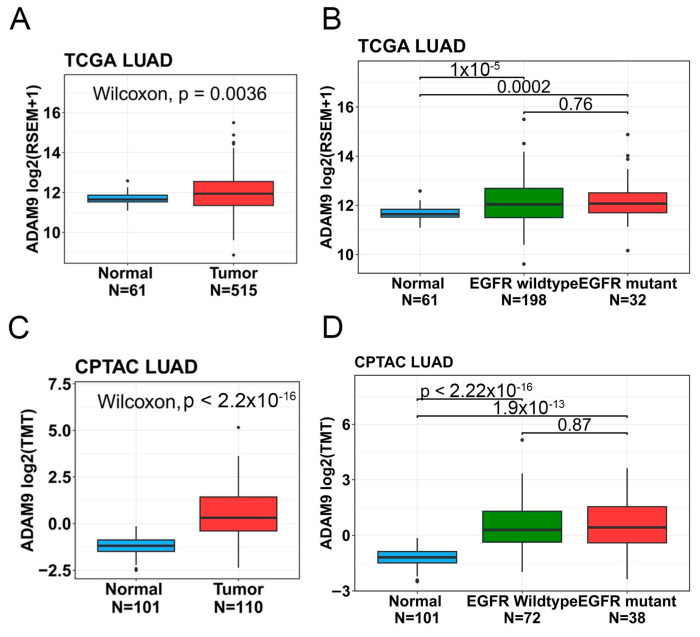
ADAM9 mRNA and protein expression levels in lung adenocarcinoma (LUAD) patients were, respectively, analyzed using data from TCGA and CPTAC databases. (**A**,**C**) ADAM9 mRNA (**A**) and protein (**C**) levels were compared between normal and tumor tissues from LUAD patients. (**B**,**D**) LUAD tissues, regardless of the *EGFR* status (wild-type or mutant), showed significantly elevated ADAM9 mRNA (**B**) and protein (**D**) levels compared to normal tissues. For multiple group comparisons, brackets have been used to indicate which groups are being compared, with *p* < 0.05 considered significant.

**Figure 3:**
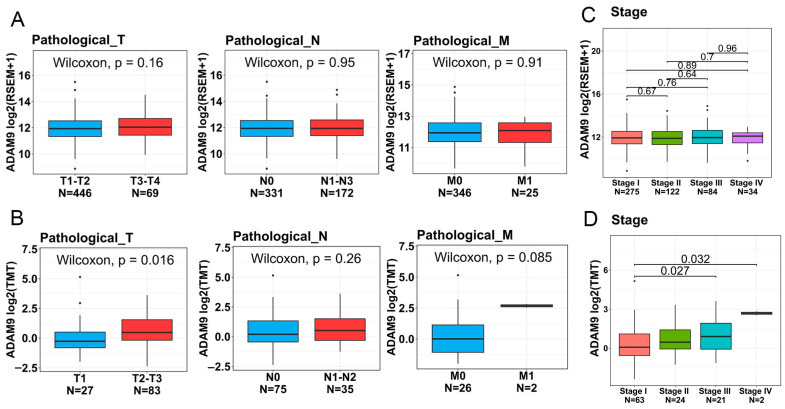
The clinical relevance of ADAM9 mRNA and protein levels in relation to clinicopathological features of lung adenocarcinoma (LUAD) patients was assessed using data from TCGA and CPTAC databases. (**A**,**C**) *ADAM9* mRNA expression levels in LUAD samples from TCGA-LUAD were analyzed based on TNM stages (**A**) and pathological stages (**C**). (**B**,**D**) ADAM9 protein expression levels in LUAD samples from CPTAC were evaluated according to TNM stages (**B**) and pathological stages (**D**). For multiple group comparisons, brackets have been used to indicate which groups are being compared, with *p* < 0.05 considered significant.

**Figure 4:**
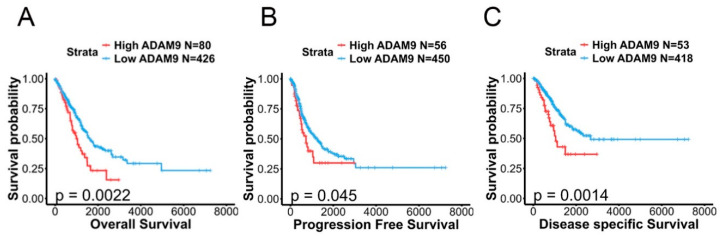
The prognostic impact of ADAM9 expression on lung adenocarcinoma (LUAD) patients was analyzed. (**A**–**C**) Kaplan–Meier survival curves depict overall survival (**A**), progression-free survival (**B**), and disease-specific survival (**C**) in LUAD patients, categorized by high or low ADAM9 expression levels. The *p*-value expresses the statistical comparison between patients with high and low ADAM9 expression. A log-rank test *p*-value < 0.05 is considered significant. The LUAD dataset was retrieved from TCGA.

To further investigate ADAM9 expression levels in both normal and LUAD tissues and their association with disease progression, we analyzed data from TCGA and CPTAC databases. The demographic characteristics of these two datasets are presented in Supplemental Tables S1 and S2. Our findings revealed that ADAM9 messenger (m)RNA and protein expression levels were significantly higher in tumor tissues compared to normal tissues (TCGA: fold change [FC] = 1.23; CPTAC: FC = 3.28; Figure 2A,C). Elevated ADAM9 mRNA and protein levels were consistently observed in LUAD tumors, regardless of whether they harbored WT or mutant EGFR; furthermore, no significant differences in ADAM9 levels were identified between tumors with WT and mutant *EGFR* (TCGA: WT vs. normal: FC = 1.31; mutant *EGFR* vs. normal: FC = 1.41; CPTAC: WT vs. normal: FC = 3.23; mutant *EGFR* vs. normal: FC = 3.38; Figure 2B,D). In addition, we observed that expression levels of the ADAM9 protein, but not mRNA, were elevated in individuals with larger tumor sizes (FC = 1.47) and distant metastases (FC = 5.14) (Figure 3A,B). Additionally, ADAM9 protein expression, but not mRNA, levels were correlated with advanced pathological stages (stage III or IV) in LUAD patients (CPTAC: stage IV vs. I: FC = 5.56; stage III vs. I: FC = 1.71; Figure 3C,D). The Kaplan–Meier survival analysis further indicated that higher ADAM9 expression levels were correlated with shorter OS (Figure 4A), PFS (Figure 4B), and DSS (Figure 4C) times.

### 2.7. Investigation of Potential Molecular Mechanisms Regulated by ADAM9 in LUAD Progression

**Figure 5:**
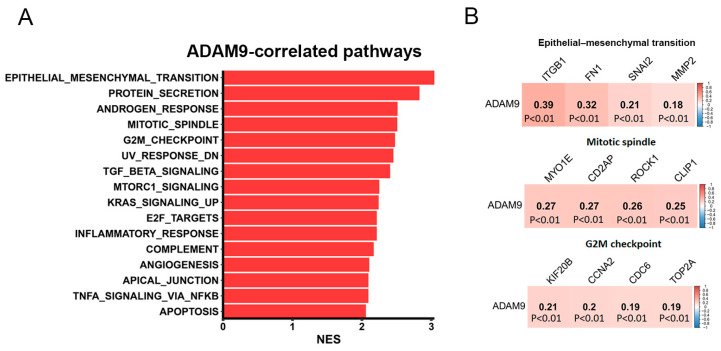
Pathways associated with ADAM9 in lung adenocarcinoma (LUAD) patients were analyzed. (**A**) A horizontal bar plot displays ADAM9-related pathways, with red bars representing pathways positively correlated with *ADAM9* expression. The X-axis shows the normalized enrichment scores (NESs), and the Y-axis lists the pathways identified from the Hallmark database. (**B**) Correlation plots illustrate the relationship between *ADAM9* expression and biomarkers of the epithelial–mesenchymal transition, mitotic spindle, and G_2_M progression. RNA-sequencing data from The Cancer Genome Atlas-LUAD dataset were used for analysis. A Pearson correlation analysis identified these associations, with correlation coefficients and *p*-values provided for each square. The scale bar reflects the strength of the correlation.

To uncover the mechanisms through which ADAM9 drives LUAD progression, we conducted a GSEA using TCGA-LUAD dataset. Our analysis revealed that epithelial–mesenchymal transition (EMT)-associated gene signatures were the top-ranked pathways correlated with *ADAM9* expression in LUAD. In addition, several proliferation-related Hallmark gene sets, such as ‘MITOTIC_SPINDLE’ and ‘G2M_CHECKPOINT’, were also observed in patients with high levels of *ADAM9* expression (Figure 5A). Analysis of human LUAD samples from TCGA using the cBioPortal platform revealed that *ADAM9* expression levels were positively correlated with mesenchymal phenotype-related genes (*FN1*, *ITGB1*, *SNAI2*, and *MMP2*), mitotic-related genes (*MYO1E*, *CD2AP*, *ROCK1*, and *CLIP1*), as well as G2/M transition-related genes (*KIF20B*, *CCNA2*, *CDC6*, and *TOP2A*) (Figure 5B).

## 3. Discussion

Recognizing the critical role of ADAM9 as a protease with oncogenic effects in cancer progression, particularly through EGFR activation in various cancers, including LUAD [17,18,26,27], we investigated polymorphisms located on promoter and intron regions of the *ADAM9* gene. These polymorphisms showed distinct distributions between LUAD patients with WT and mutant EGFR. Our findings revealed that patients carrying the mutant G allele of rs6474526 had a significantly reduced risk of developing *EGFR* mutations. Additionally, LUAD patients with at least one minor G allele of rs6474526 were found to have an increased likelihood of developing larger tumors, especially among *EGFR*-mutant, female, and non-smoking LUAD patients. Among female and non-smoking LUAD patients, a significantly increased risk of developing distant metastasis was also identified. Additionally, in patients with WT *EGFR*, the presence of a minor T allele in rs10105311 was linked to a decreased risk of progressing to advanced tumor stages.

The rs6474526 SNP is an intronic SNP of the *ADAM9* gene. While intronic polymorphisms typically do not affect protein sequences and were once regarded as junk DNA, growing evidence now highlights the significance of introns. They play critical roles in modulating gene expressions, mRNA splicing, export, and transcription coupling, and increasing protein diversity through alternative splicing and exon shuffling [28,29,30]. Moreover, introns are frequently more prone to mutations or may act as mutational hotspots due to the presence of various essential functional elements, such as intron splice silencers, enhancers, trans-splicing elements, and other regulatory components. Beyond functional mutations, SNPs within introns can also heighten disease susceptibility, such as in cancers, and influence relationships between genotypes and phenotypes [28]. For instance, the rs12203592 intronic polymorphism in the interferon regulatory factor 4 (*IRF4*) gene was associated with an increased risk of acute lymphoblastic leukemia. A C-to-T substitution in this variant increases *IRF4* gene expression by altering the binding affinity of the activator protein 2α transcription factor (TF) [31]. Likewise, three distinct variants (rs35054928, rs2981578, and rs45631563) within transcriptional silencer elements of the fibroblast growth factor receptor 2 (*FGFR2*) gene were identified. These variants boost silencer activity, reduce FGFR2 expression, and elevate the risk of breast cancer by increasing estrogen responsiveness [32]. Another example is rs72755295, an intronic SNP in the exonuclease 1 (*EXO1*) gene, which enhances enhancer activity, upregulates EXO1 expression, and contributes to breast cancer susceptibility [33]. In this study, we indicated the potential impact of the intronic rs6474526 SNP on *ADAM9* expression in lung tissues and whole blood of healthy individuals, as observed in the GTEx database. Moreover, LUAD cells carrying at least one minor G allele displayed higher ADAM9 levels compared to those with the TT genotype. Future studies should focus on collecting both mRNA and DNA from the same LUAD patient samples to validate the impact of the rs6474526 SNPs on *ADAM9* expression in LUAD patients. Additionally, further investigation is needed to understand the mechanisms through which this intronic SNP regulates *ADAM9* expression.

Smoking is a significant environmental factor linked to *KRAS* mutations in LUAD patients [34] and increased ADAM9 expression in individuals with chronic obstructive pulmonary disease [35]. Additionally, ADAM9 was shown to stabilize mutant KRAS in pancreatic cancers [36]. Since *EGFR* and *KRAS* mutations in NSCLC are generally considered mutually exclusive, we propose that LUAD tissues with WT *EGFR* and mutant *KRAS* may exhibit higher ADAM9 expression compared to tissues with mutant *EGFR*. Indeed, our findings suggested that LUAD patients carrying the mutant G allele of rs6474526 tended to express higher levels of ADAM9, which was associated with a reduced risk of developing *EGFR* mutations.

We further investigated the influence of *ADAM9* SNPs on the clinicopathological features of LUAD patients. Evidence from in vitro, in vivo, and clinical studies showed that ADAM9 facilitates lung tumor cell proliferation, adhesion to vascular endothelial cells, anoikis resistance, cell migration, tumor angiogenesis, growth, and metastasis in xenograft models. Additionally, elevated ADAM9 expression was linked to poor OS in lung cancer patients [12]. Consistent with these findings, we herein observed that ADAM9 mRNA and protein levels were significantly higher in LUAD tissues compared to noncancerous tissues. These elevated levels were correlated with advanced T stages, higher pathological stages, and worse prognostic outcomes. Furthermore, we observed that carriers of the rs6474526 G allele were significantly associated with larger tumors and distal metastases, suggesting that the rs6474526 SNPs in *ADAM9* may promote tumor progression by upregulating ADAM9 expression. These findings align with previous reports highlighting the association of rs6474526 SNPs with advanced T stages in prostate cancer patients [22].

Growing evidence has demonstrated that the EMT process is closely associated with tumorigenesis, angiogenesis, metastasis, and poor prognoses in LUAD [37,38]. Although EGFR signaling has been reported to induce the EMT in cancers [39], LUAD cells harboring constitutively active *EGFR* mutations tend to display epithelial characteristics compared to those with WT *EGFR*. Additionally, LUAD patients with *EGFR* mutations are generally considered to have a less-aggressive disease course, partly due to the presence of epithelial traits [40]. In our study, we observed that *ADAM9* expression was correlated with EMT-related gene signatures in TCGA-LUAD dataset and was associated with poor prognoses. Using the cBioPortal platform to analyze human LUAD samples further revealed a positive correlation between *ADAM9* expression and mesenchymal phenotype-related genes. A previous study has demonstrated that ADAM9 can induce the upregulation of the mesenchymal marker N-cadherin in LUAD cells with WT EGFR [41]. These findings suggest that ADAM9 may drive the EGFR-mediated EMT to facilitate LUAD progression, potentially playing a more prominent role in patients with WT *EGFR*. However, this hypothesis requires validation in future studies.

Several limitations of this study should be acknowledged. First, we analyzed the effects of *ADAM9* rs6474526 SNPs on *ADAM9* gene expression in whole blood and lung tissues of healthy individuals using data from the GTEx database. To confirm the influence of *ADAM9* SNPs on *ADAM9* expression in LUAD patients, future studies should simultaneously collect mRNA from OCT-embedded tissues and DNA from blood samples of LUAD patients. Second, while our study provides evidence of associations between *ADAM9* SNPs, tumor size, and *EGFR* mutation frequency in LUAD patients, we lack survival data for the recruited cohort. As a result, we are unable to assess the prognostic impact of *ADAM9* SNPs in this study. Therefore, long-term survival data should be collected to further evaluate the role of *ADAM9* SNPs in LUAD prognosis. Third, our analysis included lifestyle factors such as cigarette smoking status, as well as inherent factors like gender and age, as confounding variables. However, future studies should also incorporate treatment history and comorbidities into the logistic regression models for a more comprehensive analysis.

## 4. Materials and Methods

### 4.1. Study Populations and Ethics

A total of 535 patients with LUAD participated in this study at Chung Shan Medical University Hospital (Taichung, Taiwan). All patients had a minimum follow-up period of six months. Among them, 219 had wild-type *EGFR*, while 316 had *EGFR* mutations, including an exon 19 in-frame deletion and/or the L858R mutation in exon 21. LUAD specimens were collected following a protocol approved by the Institutional Review Board of Chung Shan Medical University Hospital (No. CS1-20144). The clinical data included patient gender, age, smoking history, TNM status, clinical stage, and cell differentiation.

### 4.2. Sequencing of EGFR from Tumor Tissues

The EGFR sequencing data of LUAD samples were processed as described in a previous report [42]. In brief, the fraction of tumor tissue for DNA extraction was macro-dissected to enrich tumor proportion from the individual OCT-embedded tissue block using a QIAamp DNA kit (Qiagen, Valencia, CA, USA). The DNA sample was first detected via a real-time polymerase chain reaction (PCR). Then, the ABI PRISM 3130XL System (Applied Biosystems, Foster City, CA, USA) was used to divide it into a wild-type *EGFR* or *EGFR* mutation group. The mutation *EGFR* was focused on the L858R mutation and the exon 19 in-frame deletion.

### 4.3. Genotyping of ADAM9 SNPs from Whole-Blood Samples

LUAD patients’ whole-blood samples were collected in tubes coated with ethylenediaminetetraacetic acid (EDTA). Following centrifugation, genomic DNA was isolated from the buffy coat using the QIAamp DNA Blood Mini Kit (Qiagen). A Nanodrop-2000 spectrophotometer (Thermo Scientific, Waltham, MA, USA) was used to determine the quality and concentration of the extracted DNA. Herein, four SNPs in the *ADAM9* gene were selected: two were in the intron region (rs6474526 and rs78451751) and two in the promoter region (rs7006414 and rs10105311). Individual TaqMan SNP probes, rs6474526 (assay ID: C_449039_10), rs7006414 (assay ID: C_28949418_10), rs78451751 (assay ID: C_105349195_10), and rs10105311 (assay ID: C_26007162_10), were used to identify the genotyping of the *ADAM9* SNPs using the ABI StepOnePlus™ RT-PCR System (Applied Biosystems). The results were analyzed using SDS version 3.0 software (Applied Biosystems), and the detailed DNA genotyping procedures were outlined in our previous study [43].

### 4.4. Bioinformatics Analysis

RNA sequencing data for LUAD patients were retrieved from The Cancer Genome Atlas (TCGA) database via UCSC Xena (https://xenabrowser.net/). Gene expression levels were normalized using RSEM and log2-transformed. Proteomics data from Clinical Proteomic Technology Assessment for Cancer (CPTAC) were obtained through LinkedOmics, with protein levels quantified using tandem mass tag (TMT) labeling and subsequent log2-transformation. For comparisons between two groups, such as tumor and normal tissues, as well as pathological TNM stages of *ADAM9* gene or protein expression, the Wilcoxon rank-sum test was used. For comparisons across multiple pathological stages, the Kruskal–Wallis test was performed, followed by a post-hoc Dunn’s test for pairwise analyses. Survival analyses, including OS, progression-free survival (PFS), and disease-specific survival (DSS) of TCGA LUAD patients with respect to ADAM9, were conducted using an optimal cutoff point determined to minimize the *p*-value. The log-rank test was applied, and results were visualized through Kaplan–Meier plots. To evaluate signaling pathways associated with *ADAM9* expression, a gene set enrichment analysis (GSEA) was conducted. Genes were ranked based on their correlation coefficients with *ADAM9* expression, and the GSEA was performed using the Hallmark gene set database. The top pathways positively correlated with *ADAM9* (with a false discovery rate (FDR) of <0.01) are presented in a horizontal bar plot. RNA expression levels of *ADAM9* in LUAD cell lines were obtained from the DepMap portal database (DepMap public 24Q4, Broad Inst.). Values are presented as transcripts per million (TPM).

### 4.5. Western Blot Analysis

Protein lysates from LUAD cells were prepared and quantified as in our previous study [44]. Equal protein amounts were subjected to SDS-PAGE, transferred to PVDF membranes, and probed with primary antibodies for ADAM9 (no.2099; Cell Signaling Technology, Danvers, MA, USA) and GAPDH (60,004–1-Ig; Proteintech, Chicago, IL, USA). After three TBST washes, membranes were incubated with a secondary antibody at room temperature, and protein detection was performed using chemiluminescence.

### 4.6. Statistical Analysis

The chi-squared test and Student’s *t*-test were utilized to compare demographic characteristics between patients with WT *EGFR* and those with mutant *EGFR*. Multiple logistic regression models were applied to calculate odds ratios (ORs) and adjusted ORs (AORs), along with their 95% confidence intervals (CIs), to assess associations of genotypic frequencies and *EGFR* status groups with the risk of various clinicopathological characteristics. Confounding factors include lifestyle variables such as smoking and alcohol consumption, as well as inherent factors like gender and age. All statistical analyses were conducted using SAS software (version 9.1, 2005 release for Windows; SAS Institute, Cary, NC, USA), with a significance threshold set at *p* < 0.05.

## 5. Conclusions

In summary, this is the first study to explore distinct allelic effects of *ADAM9* SNPs in a Taiwanese population, highlighting their impacts on *EGFR* mutation frequency and tumor progression in LUAD. Our findings demonstrated that rs6474526 intronic SNPs are associated with *EGFR* mutation susceptibility. Moreover, both the rs6474526 intronic SNPs and the rs10105311 promoter SNPs show promise as potential markers for predicting disease progression in LUAD patients. Additionally, our results suggest that ADAM9-related pathways, such as the EMT, may act as key drivers of LUAD progression. Predicting cancer risk and disease progression through SNP analysis from non-invasive biopsies offers valuable insights for personalized medicine. Our findings revealed that rs6474526 variants were significantly associated with larger tumor sizes and distant metastases of LUAD in female and non-smoking groups, suggesting that this *ADAM9* SNP may serve as a useful predictor of disease progression in both LUAD populations.

## Figures and Tables

**Table 1:** Demographics and clinical characteristics of 535 lung adenocarcinoma patients with the *EGFR* mutation status.

Variable	*EGFR* Wild-Type (*N* = 219) *n* (%)	*EGFR* Mutation (*N* = 316) *n* (%)	*p*-Value
Age (years)			
Mean ± SD	64.63 ± 12.23	65.36 ± 12.51	*p* = 0.523
Gender			
Male	134 (61.2%)	105 (33.2%)	*p* < 0.001
Female	85 (38.8%)	211 (66.8%)	
Cigarette smoking status			
Never-smoker	103 (47.0%)	255 (80.7%)	*p* < 0.001
Ever-smoker	116 (53.0%)	61 (19.3%)	
Stage			
I or II	48 (21.9%)	76 (24.1%)	*p* = 0.565
III or IV	171 (78.1%)	240 (75.9%)	
Tumor T status			
T1 or T2	108 (49.3%)	171 (54.1%)	*p* = 0.275
T3 or T4	111 (50.7%)	145 (45.9%)	
Lymph node status			
Negative	59 (26.9%)	89 (28.2%)	*p* = 0.756
Positive	160 (73.1%)	227 (71.8%)	
Distant metastasis			
Negative	97 (44.3%)	126 (39.9%)	*p* = 0.308
Positive	122 (55.7%)	190 (60.1%)	
Cell differentiation			
Well	18 (8.2%)	35 (11.1%)	*p* < 0.001
Moderately	136 (62.1%)	248 (78.5%)	
Poorly	65 (29.7%)	33 (10.4%)	

**Table 2:** Distribution frequencies of *ADAM9* genotypes in lung adenocarcinoma patients and multivariate logistic regression analysis of their associations with *EGFR* mutations.

Genotypes	*EGFR* Wild-Type (*N* = 219)	*EGFR* Mutation (*N* = 316)	AOR (95% CI)	*p*-Value
rs6474526				
TT	192 (87.7%)	296 (93.7%)	1.000	
TG	27 (12.3%)	20 (6.3%)	0.477 (0.260~0.875)	*p* = 0.017 *
GG	0 (0.0%)	0 (0.0%)	---	---
TG + GG	27 (12.3%)	20 (6.3%)	0.477 (0.260~0.875)	*p* = 0.017 *
rs7006414				
TT	136 (62.1%)	198 (62.7%)	1.000	
TC	67 (30.6%)	102 (32.3%)	1.156 (0.769~1.738)	*p* = 0.485
CC	16 (7.3%)	16 (5.0%)	0.652 (0.302~1.412)	*p* = 0.278
TC + CC	83 (37.9%)	118 (37.3%)	1.051 (0.717~1.540)	*p* = 0.799
rs10105311				
CC	155 (70.8%)	219 (69.3%)	1.000	
CT	56 (25.6%)	83 (26.3%)	1.073 (0.700~1.644)	*p* = 0.746
TT	8 (3.6%)	14 (4.4%)	1.017 (0.397~2.607)	*p* = 0.972
CT + TT	64 (29.2%)	97 (30.7%)	1.065 (0.711~1.595)	*p* = 0.759
rs78451751				
TT	216 (98.6%)	314 (99.4%)	1.000	
TC	3 (1.4%)	2 (0.6%)	0.372 (0.058~2.408)	*p* = 0.300
CC	0 (0.0%)	0 (0.0%)	---	---
TC + CC	3 (1.4%)	2 (0.6%)	0.372 (0.058~2.408)	*p* = 0.300

Adjusted odds ratios (AORs) and 95% confidence intervals (CIs) were calculated using multiple logistic regression models, with adjustments for age, gender, and cigarette smoking status. * A *p* value of < 0.05 was defined as statistically significant.

**Table 3:** Clinicopathological characteristics of lung adenocarcinoma patients with wild-type or mutant *EGFR*, stratified by the polymorphic genotypes of *ADAM9* rs6474526.

Variable	All (*N* = 535)			*EGFR* Wild-Type (*N* = 219)			*EGFR* Mutation (*N* = 316)		
	TT (*N* = 488)	TG + GG (*N* = 47)	*p*-Value	TT (*N* = 192)	TG + GG (*N* = 27)	*p*-Value	TT (*N* = 296)	TG + GG (*N* = 20)	*p*-Value
Stage									
I or II	116 (23.8%)	8 (22.8%)	*p* = 0.295	42 (21.9%)	6 (22.2%)	*p* = 0.967	74 (25.0%)	2 (10.0%)	*p* = 0.129
III or IV	372 (76.2%)	39 (83.0%)		150 (78.1%)	21 (77.8%)		222 (75.0%)	18 (90.0%)	
Tumor T status									
T1 or T2	262 (53.7%)	17 (36.2%)	*p* = 0.022 ^a^	97 (50.5%)	11 (40.7%)	*p* = 0.341	165 (55.7%)	6 (30.0%)	*p* = 0.025 ^b^
T3 or T4	226 (46.3%)	30 (63.8%)		95 (49.5%)	16 (59.3%)		131 (44.3%)	14 (70.0%)	
Lymph node status									
Negative	138 (28.3%)	10 (21.3%)	*p* = 0.305	52 (27.1%)	7 (25.9%)	*p* = 0.899	86 (29.1%)	3 (15.0%)	*p* = 0.176
Positive	350 (71.7%)	37 (78.7%)		140 (72.9%)	20 (74.1%)		210 (70.9%)	17 (85.0%)	
Distant metastasis									
Negative	208 (42.6%)	15 (31.9%)	*p* = 0.155	87 (45.3%)	10 (37.0%)	*p* = 0.418	121 (40.9%)	5 (25.0%)	*p* = 0.160
Positive	280 (57.4%)	32 (68.1%)		105 (54.7%)	17 (63.0%)		175 (59.1%)	15 (75.0%)	
Cell differentiation									
Well/Moderately	403 (82.6%)	34 (72.3%)	*p* = 0.083	137 (71.4%)	17 (63.0%)	*p* = 0.372	266 (89.9%)	17 (85.0%)	*p* = 0.491
Poorly	85 (17.4%)	13 (27.7%)		55 (28.6%)	10 (37.0%)		30 (10.1%)	3 (15.0%)	

^a^ Odds ratio (OR) (95% confidence interval (CI)): 2.046 (1.099–3.807), ^b^ OR (95% CI): 2.939 (1.099–7.858).

**Table 4:** Clinicopathological characteristics of lung adenocarcinoma patients with wild-type or mutant *EGFR*, stratified by the polymorphic genotypes of *ADAM9* rs10105311.

Variable	All (*N* = 535)			*EGFR* Wild-Type (*N* = 219)			*EGFR* Mutation (*N* = 316)		
	CC (*N* = 374)	CT + TT (*N* = 161)	*p*-Value	CC (*N* = 155)	CT + TT (*N* = 64)	*p*-Value	CC (*N* = 219)	CT + TT (*N* = 97)	*p*-Value
Stage									
I or II	82 (21.9%)	42 (26.1%)	*p* = 0.295	28 (18.1%)	20 (31.2%)	*p* = 0.032 ^a^	54 (24.7%)	22 (22.7%)	*p* = 0.704
III or IV	292 (78.1%)	119 (73.9%)		127 (81.9%)	44 (68.8%)		165 (75.3%)	75 (77.3%)	
Tumor T status									
T1 or T2	190 (50.8%)	89 (55.3%)	*p* = 0.342	74 (47.7%)	34 (53.1%)	*p* = 0.469	116 (53.0%)	55 (56.7%)	*p* = 0.539
T3 or T4	184 (49.2%)	72 (44.7%)		81 (52.3%)	30 (46.9%)		103 (47.0%)	42 (43.3%)	
Lymph node status									
Negative	102 (27.3%)	46 (28.6%)	*p* = 0.758	36 (23.2%)	23 (35.9%)	*p* = 0.054	66 (30.1%)	23 (23.7%)	*p* = 0.241
Positive	272 (72.7%)	115 (71.4%)		119 (76.8%)	41 (64.1%)		153 (69.9%)	74 (76.3%)	
Distant metastasis									
Negative	149 (39.8%)	74 (46.0%)	*p* = 0.188	63 (40.6%)	34 (53.1%)	*p* = 0.091	86 (39.3%)	40 (41.2%)	*p* = 0.742
Positive	225 (60.2%)	87 (54.0%)		92 (59.4%)	30 (46.9%)		133 (60.7%)	57 (58.8%)	
Cell differentiation									
Well/Moderately	306 (81.8%)	131 (81.4%)	*p* = 0.901	113 (72.9%)	41 (64.1%)	*p* = 0.193	193 (88.1%)	90 (92.8%)	*p* = 0.212
Poorly	68 (18.2%)	30 (18.6%)		42 (27.1%)	23 (35.9%)		26 (11.9%)	7 (7.2%)	

^a^ Odds ratio (95% confidence interval): 0.485 (0.249–0.946).

**Table 5:** *ADAM9* rs6474526 genotype distributions and clinicopathological characteristics of lung adenocarcinoma patients who are female or non-smokers.

Variable	Female (*N* = 296)				Non-Smoker (*N* = 358)			
	TT (*N* = 278)	TG + GG (*N* = 18)	OR (95% CI)	*p*-Value	TT (*N* = 336)	TG + GG (*N* = 22)	OR (95% CI)	*p*-Value
Stage								
I or II	73 (26.3%)	2 (11.1%)	1.000	*p* = 0.152	87 (25.9%)	2 (9.1%)	1.000	*p* = 0.077
III or IV	205 (73.7%)	16 (88.9%)	2.849 (0.639~12.692)		249 (74.1%)	20 (90.9%)	3.494 (0.800~15.256)	
Tumor T status								
T1 or T2	156 (56.1%)	4 (22.2%)	1.000	*p* = 0.005	185 (55.1%)	4 (18.2%)	1.000	*p* = 0.001
T3 or T4	122 (43.9%)	14 (77.8%)	4.475 (1.437~13.940)		151 (44.9%)	18 (81.8%)	5.513 (1.827~16.638)	
Lymph node status								
Negative	89 (32.0%)	3 (16.7%)	1.000	*p* = 0.173	101 (30.1%)	4 (18.2%)	1.000	*p* = 0.236
Positive	189 (68.0%)	15 (83.3%)	2.354 (0.665~8.342)		235 (69.9%)	18 (81.8%)	1.934 (0.639~5.858)	
Distant metastasis								
Negative	118 (42.4%)	3 (16.7%)	1.000	*p* = 0.031	140 (41.7%)	3 (13.6%)	1.000	*p* = 0.009
Positive	160 (57.6%)	15 (83.3%)	3.688 (1.044~13.029)		196 (58.3%)	19 (86.4%)	4.524 (1.313~15.583)	
Cell differentiation								
Well/ Moderately	243 (87.4%)	14 (77.8%)	1.000	*p* = 0.242	288 (85.7%)	16 (72.7%)	1.000	*p* = 0.099
Poorly	35 (12.6%)	4 (22.2%)	1.984 (0.618~6.368)		48 (14.3%)	6 (27.3%)	2.250 (0.839~6.036)	

OR, odds ratio; CI, confidence interval.

## Data Availability

The data from this study are available upon reasonable request to the corresponding author.

## Supplementary Materials

The following supporting information can be downloaded at https://www.mdpi.com/article/10.3390/ijms26104606/s1.

## Abbreviations

ADAM9: A disintegrin and metalloproteinase 9; AOR: adjusted odds ratio; CI: confidence interval; CPTAC: Clinical Proteomic Tumor Analysis Consortium; DSS: disease-specific survival; EGFR: epidermal growth factor receptor; EMT: epithelial–mesenchymal transition; GSEA: gene set enrichment analysis; GTEx: Genotype-Tissue Expression; GWAS: genome-wide association study; LUAD: lung adenocarcinoma; NSCLC: non-small-cell lung cancer; OS: overall survival; PFS: progression-free survival; RT-PCR: real-time polymerase chain reaction; SNP: single-nucleotide polymorphism; TCGA: The Cancer Genome Atlas; TNM: tumor/node/metastasis; WT: wild-type.
