# Supplementary material for: Potential Influence of ADAM9 Genetic Variants and Expression Levels on the EGFR Mutation Status and Disease Progression in Patients with Lung Adenocarcinoma
Source: Int J Mol Sci. 2025 May 11;26(10):4606. doi: 10.3390/ijms26104606 (PMC12111331; doi:10.3390/ijms26104606)
Supplement: Supplementary file 1 [file ijms-26-04606-s001.zip › ijms-3583664-supplementary.pdf]

Supplemental Information

Title:

Potential influence of ADAM9 genetic variants and expression levels on the EGFR mutation status and disease progression in patients with lung adenocarcinoma

Jer-Hwa Chang, Tsung-Ching Lai, Kuo-Hao Ho, Thomas Chang-Yao Tsao, Lun-Ching Chang, Shun-Fa Yang, Ming-Hsien Chien

Correspondence to: Dr. Shun-Fa Yang (E-mail: ysf@csmu.edu.tw) and Dr. Ming-Hsien Chien (E-mail: mhchien1976@gmail.com)

Table S1. Demographics and clinical characteristics of 230 lung adenocarcinoma patients with the EGFR mutation status from TCGA

**Variable**	**EGFR wild type (*N*=198) *n* (86.1%)**	**EGFR mutation (*N*=32) *n* (13.9%)**	***p* value**
**Age (years)**			
Mean ± SD	65.21 ± 9.95	67.26 ± 9.69	*p*=0.779
**Gender**			
Male	89 (44.9%)	10 (31.3%)	*P*=0.207
Female	109 (55.1%)	22 (68.8%)	
**Cigarette smoking status**			
Never-smoker	21 (11.6%)	10 (33.3%)	*P*=0.005
Ever-smoker	160 (88.4%)	20 (66.6%)	
**Stage**			
I or II	147 (74.2%)	24 (75.0%)	*p*=1.000
III or IV	51 (25.8%)	8 (25.0%)	
**Tumor T status**			
T1 or T2	171 (86.4%)	27 (84.4%)	*p*=0.979

T3 or T4	27 (13.6%)	5 (15.6%)	
**Lymph node status**			
Negative	122 (63.2%)	17 (56.7%)	*p*=0.627
Positive	71 (36.8%)	13 (43.3%)	
**Distant metastasis**			
Negative	146 (95.4%)	27 (96.4%)	*p*=1.000
Positive	7 (4.6%)	1 (3.6%)	

Table S2. Demographics and clinical characteristics of 230 lung adenocarcinoma patients with the EGFR mutation status from CPTAC

**Variable**	**EGFR wild type (*N*=72) *n* (65.5%)**	**EGFR mutation (*N*=38) *n* (34.5%)**	***p* value**
**Age (years)**			
Mean ± SD	67.16 ± 7.61	68.86 ± 7.29	*p*=0.800
**Gender**			
Male	53 (73.6%)	19 (50.0%)	*P*=0.023
Female	19 (26.4%)	19 (50.0%)	
**Cigarette smoking status**			
Never-smoker	23 (32.9%)	23 (62.2%)	*P*=0.006
Ever-smoker	47 (67.1%)	14 (37.8%)	
**Stage**			
I or II	59 (81.9%)	28 (73.7%)	*p*=0.443
III or IV	13 (18.1%)	10 (26.3%)	
**Tumor T status**			
T1 or T2	65 (90.3%)	32 (84.2%)	*p*=0.531
T3 or T4	7 (9.7%)	6 (15.8%)	
**Lymph node status**			
Negative	54 (75.0%)	21 (55.3%)	*p*=0.057
Positive	18 (25.0%)	17 (44.7%)	
**Distant metastasis**			
Negative	19 (90.5%)	7 (100.0%)	*p*=1.000
Positive	2 (9.5%)	0 (0%)	
